# *“The best thing is that you are doing it for yourself” –* perspectives on acceptability and feasibility of HPV self-sampling among cervical cancer screening clients in Tanzania: a qualitative pilot study

**DOI:** 10.1186/s12905-020-00917-7

**Published:** 2020-03-31

**Authors:** Aleksandra Bakiewicz, Vibeke Rasch, Julius Mwaiselage, Ditte S. Linde

**Affiliations:** 1 Department of Clinical Research, University of Southern Denmark, 5000 Odense C, Denmark; 2 Department of Gynaecology and Obstetrics, Odense University Hospital, Odense, Denmark; 3 Department of Cancer Prevention Services, Ocean Road Cancer Institute, Dar es Salaam, Tanzania; 4 OPEN, Odense Patient Data Explorative Network, Odense University Hospital, Odense, Denmark

**Keywords:** Self-sampling, Cervical screening, HPV, Acceptability, Feasibility, Qualitative study, Tanzania

## Abstract

**Background:**

Cervical cancer is the most common type of cancer in sub-Saharan Africa, and it is also the cancer disease that most women die from. The high mortality rate is partly due to low attendance rates to screening services and low sensitivity of visual inspection with acetic acid, which is the standard screening method used in screening programs in sub-Saharan Africa. In order to overcome of the burden of disease new screening strategies and methods are warranted. This study aims to explore the acceptability and feasibility of HPV self-sampling compared to provider-based sampling among cervical cancer screening clients living in Dar es Salaam.

**Methods:**

Women attending cervical cancer screening at Ocean Road Cancer Institute in Dar es Salaam, Tanzania between February – April 2017 were invited into the study. The participants had (1) a provider-collected sample, and (2) a self-sample for HPV on top of the regular cervical cancer screening. 50% of the participants conducted the self-sample after receiving a written instruction guide of how to collect the sample (written). The other 50% received both the written and an oral introduction to self-sampling (written+). All participants could ask for nurse assistance during self-sample collection if needed. Individual semi-structured interviews were conducted with the participants post sample collection. Data collection stopped when saturation was reached. Data were analysed using a thematic content analysis.

**Results:**

Twenty-one women participated in the study. Regardless of how women were introduced to the self-sample (written or written+), there was a high demand for nurse presence as they felt uncertain of their personal capabilities to collect the self-sample correctly. However, as long as nurse assistance was an option most women perceived self-sampling as easy and comfortable though few experienced bleeding and pain. The majority of women preferred self-sampling over provider-sampling primarily due to the method being more private than the provider-sampling.

**Conclusions:**

HPV self-sampling was well-perceived and accepted, however, for the method to be feasible a nurse needed to be present. HPV Self-sampling may be an alternative method to increase uptake of cervical cancer screening. Larger quantitative studies are recommended to support the study findings.

## Background

Despite cervical cancer being the most treatable and preventable form of cancer if detected early and treated properly, the disease is the fourth most common cancer among women worldwide [1]. Every year more than half a million women develop cervical cancer of which almost 50% die (*n* = 265,700). Inequity for cervical cancer is high as 87% of all cases occur in less developed regions of the world [2]. Cervical cancer is caused by persistent high-risk Human Papillomavirus (HR HPV) infection, and although HPV vaccination holds promise to prevent the disease in a long term perspective, cervical screening is a necessary supplementary method as the current vaccine does not protect against all HR HPV types and is yet to be fully implemented in many low-income countries [3]. In addition, vaccination programs mainly target school girls, and therefore the world currently faces a whole generation of women that fully rely on screening. Finally, many women from low-income settings lack knowledge of the risk of the disease and how to prevent it [4].

Cervical cancer is the most common cancer among Tanzanian women. It constitutes 38.4% of all newly developed cancers and it is the main cause of female cancer deaths (34.3%) [5]. The age-standardised incidence rate (ASRs) is 59.1 per 100,000 Tanzanian women, which is almost double the average ASR for Africa (27.6 per 100,000 women) [4, 5]. The major burden of disease is partly due to poor coverage (4–6%) of cervical screening programs [6, 7] and those women who do attend screening often come when symptoms are present and the disease has progressed to advanced stages [8]. A recent systematic review of cervical cancer in Tanzania found that this is due to a number of factors including fear, stigma, unawareness of screening options as well as costs related to screening [9, 10]. Therefore, innovative ways of how to improve cervical cancer screening attendance is needed.

In Tanzania, the standard screening method is visual inspection with acid (VIA), which requires a gynaecological examination [4]. However, studies have questioned the sensitivity and specificity of VIA [11–13] and therefore rapid HPV testing is currently being tested as a promising primary method for cervical cancer screening. HPV testing usually requires a gynaecological examination, however, a vaginal sampling can also be taken by the woman herself, which is referred to as HPV self-sampling or self-testing. HPV self-sampling is considered as specific as provider-sampling and thus an acceptable alternative [14–16]. There are different devices of HPV self-sampling available in the market, for example, Qvintip [12], the Evalyn Brush, the Viba Brush and the Delphi Screener [17, 18].

Research conducted in both low- and high-income countries generally suggest good feasibility and acceptability of HPV self-sampling. European studies have shown that women from Germany (89%), Netherlands (91%) and Italy (94%) perceived self-sampling as easy [19–21], and women from the Netherlands (75%), Italy (78%) and the US (79%) preferred self-sampling over a provider-based examination [21–23]. Research on feasibility and acceptability of HPV self-sampling in Africa is limited, however a few studies have been conducted in Rwanda, Uganda, Kenya, South Africa, Cameroon, and Nigeria [24–33]. Overall, self-sampling was found to be an acceptable and feasible screening method. For example, questionnaire surveys from Rwanda and Uganda showed that 70 and 80% of women preferred self-sampling over provider-sampling [24, 26] and mixed method studies from Rwanda, South Africa, and Nigeria found it to be a feasible method [24, 29, 33]. However, some studies also reported concerns in relation to self-sampling, i.e. not trusting the HPV results [32] as well as feeling nervous, insecure, or inexperienced [24]. To the authors’ knowledge, there is no qualitative study of how Tanzanian women perceive HPV self-sampling.

### Aim

The objective of this study is to investigate the feasibility and acceptability of HPV self-sampling among Tanzanian women who attended a patient-initiated cervical cancer screening compared to provider-based HPV sampling.

### Study context

This qualitative study is a sub-study of a larger research project called CONCEPT (Comprehensive Cervical Cancer Prevention in Tanzania), which is a five-year international project (2015–2019) between Ocean Road Cancer Institute (ORCI), Kilimanjaro Christian Medical Centre (KCMC), The Danish Cancer Society and University of Southern Denmark (SDU). The aim of CONCEPT is to improve cervical cancer prevention in Tanzania, and it has multidisciplinary sub-studies [10, 11, 34] including establishment of a cohort of 4000 women in order to get regional-specific data on the natural history of cervical cancer and investigate the potential of HPV testing as a primary screening method.

## Methods

### Participants

Women who attended a patient-initiated screening and were included into the CONCEPT study were eligible for inclusion into this study. Inclusion criteria for the CONCEPT study were women aged 25–60 years, and exclusion criteria were pregnancy or menstruation on day of enrolment, previous hysterectomy, cervical cancer or diagnosis of cervical precancerous lesions within the past 12 months. A non-random purposive sampling technique was used with the aim to cover women of various age groups. The reason for this was to see if feasibility and acceptability was perceived differently by different age groups. Women were asked if they wanted to participate in the study by a screening nurse. The study procedure was explained to eligible participants and all participants consented to having samples collected and being interviewed. They all signed a consent form prior to the study starting.

### Data collection

In order to get a user perspective of how screening clients perceived HPV self-sampling as compared to provider-based sampling, 21 individual interviews - including two pre-tests - were conducted at the ORCI in Dar es Salaam, Tanzania after the participants had had a provider-based sample and self-sample collected. Data were collected between 21 February – 27 April 2017, and data collection stopped once saturation had been reached. The first author, who is a female, and lived in Tanzania for a longer period of time, interviewed all participants. Seventeen interviews were conducted in Swahili with simultaneous translation into English, and four interviews were conducted in English. The translator was a Tanzanian female social worker and experienced translator from ORCI.

The interviews were conducted in a private room at ORCI shortly after the woman had conducted the HPV self-sampling and had had a provider-based sample collected on top of a regular cervical cancer screening, which entailed a gynaecologic examination and VIA. The results of the HPV tests were given to the participants once the samples had been processed in the laboratory at ORCI. The interviews took place between 9 am – 4 pm and lasted between 18 to 39 min, with an average duration of 27 min. No other persons were present during the interview room except the participant, the primary investigator and the translator. Each interview was audio-recorded with the consent of the participants. Each interview was debriefed once finished.

### Instruments

**Fig. 1:**
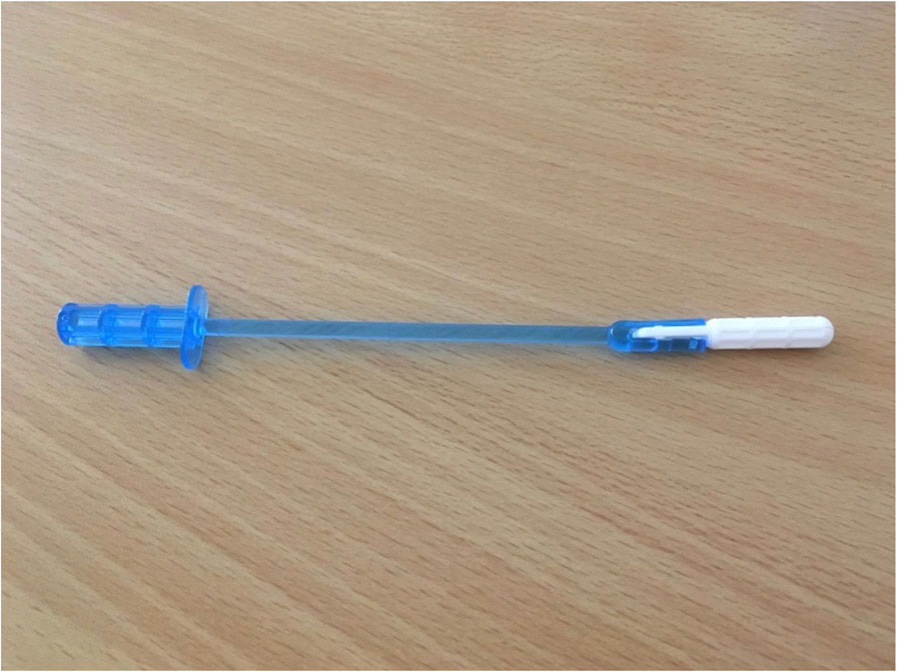
The self-sampling device used in the study - *Care*HPV *Qvintip*

In order to understand how self-confident the participants felt collecting the self-sample and the importance of health provider guidance, 50% of the participants (10 women) received both a written instruction guide with illustrations (Additional file 1) of how to collect the self-sample as well as an oral instruction provided by a nurse (written+). The other 50% (11 women) only received the written instruction guide (written). However, for ethical reasons both groups could ask for nurse assistance during the self-sampling at any time if needed. However if comfortable, the women were encouraged to try and do it on their own to see if they would be able to do the test in a non-clinical setting without a nurse being present. The written instruction guide of how to collect the self-sample was developed by a Tanzanian medical doctor. The guide was developed in English and translated into Swahili. Additionally, screening nurses were trained in the self-sampling procedure and in how to assists participants if needed. The device used for the study was the *Care*HPV Qvintip (Fig. 1) [17].

The instrument used for the interviews was a semi-structured interview guide, which was developed by the first author. The final interview guide consisted of 29 questions and the domain of interests were attitudes and experiences with self-sampling, ability to conduct the self-sampling, preferences and concerns (Additional file 2). It was inspired by other qualitative studies within the field of HPV self-sampling [25, 35, 36]. The guide was slightly modified during the first interviews. The research paradigm that guided this study was social constructivism where reality is seen as culturally and socially constructed. Hence, the knowledge that was gained in this study was subjective rather than objective and generated through the interaction between the researcher and participants [37].

### Data analysis

All audio-recorded interviews were transcribed verbatim by the first author and sounds, pauses and interruptions during interviews were noted in the transcripts. A thematic content analysis was carried out where transcripts were colour coded and labelled according to the themes that arose from the material in an inductive manner. Initial themes were discussed with a peer researcher in order to increase validation, which led to a re-categorisation of some themes. Two overall themes were part of the final analysis, namely (1) feasibility and (2) acceptability. By feasibility, we understand women’s abilities to conduct the HPV self-sampling correctly and what difficulties they experience during the self-sampling. This theme was sub-divided into two sub-themes: (1) difficulties and (2) symptoms. By acceptability, we understand women’s perception/attitude towards HPV self-sampling. This theme was further sub-divided into the following sub-themes: (1) privacy, (2) nurse presence, and (3) preferences (HPV self-sampling vs. HPV provider-sampling) (Table 1). In order to avoid the translation link, “she” was replaced with “I” in all transcripts, and each transcript ID was replaced by a fake name. The top twenty-one female Tanzanian names were used. The study is reported according to the SRQR guidelines which is a standard tool for reporting qualitative research [38].

**Table 1:** Study themes and sub-themes

Study themes	Feasibility	Acceptability
Sub-themes	• Difficulties • Symptoms	• Privacy • Nurse presence • Preferences

## Results

### Characteristics of participants

A total of 21 women participated in the study (Table 2). All women were from the Dar es Salaam Region in Tanzania. The women’s age ranged from 26 to 52 years with a mean of 39 years – the mean was 42 years in the group that had the written instructions (written) and 36 years in the group that had the verbal and oral instructions (written+). Overall, the education level varied among participants. Half of the participants had finished primary education and four had graduated from university. One woman in the study was illiterate, hence she only benefitted from the graphical and oral instruction. Almost all women were married, except for three and one was a widow. Slightly more women were Christians (57%) than Muslims. More than half of the women had more than three children. One woman in the study was HIV-positive. Two women tested HPV-positive based on the self-samplings, which was concordant with the results from the provider-collected HPV tests.

**Table 2:** Characteristics of study participants

Sociodemographic characteristics	Number (%)
**Age**	
Mean	39
26–29	2 (10%)
30–39	11 (52%)
40–49	5 (24%)
50–52	3 (14%)
**Education**	
Primary	10 (48%)
Secondary	7 (33%)
University	4 (19%)
**Marital status**	
Married	17 (81%)
Single	3 (14%)
Widow	1 (5%)
**Religion**	
Christian	12 (57%)
Muslim	8 (38%)
Other	1 (5%)
**No of pregnancies**	
0	2 (10%)
1–2	7 (32%)
3–4	6 (29%)
5–6	6 (29%)
**HIV status**	
positive	1 (5%)
negative	20 (95%)
**HPV status**	**Provider-sampling** **——————** **Self-sampling**
positive	2 (10%) **——————** 2 (10%)
negative	19 (90%) **——————** 19 (90%)
**Total number of women**	21 (100%)

### Feasibility

#### Difficulties

During the interviews, all women were asked to describe how they conducted the self-sampling and they could illustrate their point of views by using a copy of the device, which was present on the table in the interview room. For example, Jesca explained the self-sampling this way,

[...] [In] the first step I had to [...] open the package with the test. I took off my pants and put it inside. I cycled two times, removed it and put it in the container. I was told to put the sample in the container and to never touch the white place of the device. After I finished, I put it in the container. (Jesca; assigned written/did not ask for nurse guidance)

Most of the women explained the procedure quite well, however, only two women finished the self-sampling without any form of nurse assistance. Hence, nine out of the 11 women, who had initially only received a written instruction, asked for further assistance from a nurse. Furthermore, despite receiving nurse assistance, only three women conducted the self-sampling 100% correctly; one woman had received a written instruction and two had received written+ (Table 3). Further, ten women forgot to wait 3 min before putting the sample into the container as they were instructed to; six women did not wait 3 min and failed at putting the sample into the container; and two women failed at putting the sample into the container. However, even though all the steps of how to do the self-sampling were not conducted 100% correctly, the HPV test results were concordant with the provider-collected samples (Table 2).

**Table 3:** Ability to conduct the self-sampling

Self-sampling feasibility	Written instruction/ completed self-sample without nurse assistance	Written instruction/ asked for nurse assistance to complete self-sample	Written + oral instruction/ completed self-sample without further nurse assistance	Written + oral instruction/ asked for further nurse assistance to complete self-sample	***Tota****l*
Self-sampling 100% correctly	1	–	2	*–*	***3***
Did not wait 3 min (step 4)	1	9	.-	*–*	***10***
Did not wait 3 min (step 4) and failed at putting the sample into a container (step 5)	–	–	–	6	***6***
Failed at putting the sample into a container (step 5)	–	–	2	*–*	***2***
***Total***	***2***	***9***	***4***	***6***	***21***

Many women found it hard to put the self-collected sample into the container as this involved breaking off the white part of the self-collected sample device before putting it into the container. Also, the women described difficulties of inserting the device into vagina and opening the container with the storage medium. Khadija described her challenges in this way,

It was difficult to put it in the container. It will be also difficult for other women to put this in this container [...] (Khadija; assigned written+/asked for nurse guidance).

While Joan said that,

[…] [It] was hard was to insert it [ed. the self-sample brush] in the vagina - it is too long. To insert it was a bit hard. (Joan; assigned written/asked for nurse guidance).

#### Symptoms

Two women experienced a bit of bleeding during self-sampling, and three experienced pain. However, bleeding was stated by Aneth to be due to an ongoing period,

I am on my period – the last day, so I had a little bit of blood but it’s not because of the test.

(Aneth; assigned written+/did not ask for nurse guidance)

However, Khadija said that, “*[...] I am not on my period and I was bleeding* “(Khadija; assigned written+/asked for nurse guidance) and it seemed clear that she felt uncomfortable about collecting the self-sample. Another symptom that was experience by Jesca was “pain”,

I was told that I had to put it inside until it stops. After I put it inside to reach that point I felt a little bit of pain. (Jesca; assigned written/did not ask for nurse guidance).

### Acceptability

Most of the women who attended patient-initiated screening found HPV self-sampling an acceptable method due to privacy, however only with a nurse being present. In addition, most of them preferred HPV self-sampling compared to provider-sampling.

#### Privacy

All women, except for one (Khadija), expressed positive attitudes towards self-sampling and found it acceptable even though some also felt scared at first when seeing the device. For example, Jesca said that,

[...] The first time I she saw this [ed. the self-sampling device], I was thinking: “This is what?! Do I have to put it inside?! [...] I was afraid. (Jesca; assigned written/did not ask for nurse guidance).

But after collecting the self-sample most women felt comfortable. Mary said, “*I was feeling happy, very comfortable, and peaceful*” (Mary; assigned written+/did not ask for nurse guidance). Miriam described the procedure this way,

It is comfortable. I don’t think I would change anything. Very comfortable.

(Miriam; assigned written/asked for nurse guidance)

The main reason why the women liked self-sampling was due to privacy. The word *privacy* was used 40 times during all interviews in relation to the sampling process. Lillian said that,

The best thing [ed. about the self-sampling] is that you are doing it for yourself. [...] By doing it for yourself it feels comfortable because it is you. (Lillian; assigned written+/did not ask for nurse guidance).

While Jesca said,

You are doing this test [ed. self-sampling] just alone - yourself, and the normal procedure is done by the nurse [...]. I am afraid, and I feel shy when there is more than one person around.

(Jesca; assigned written/did not ask for nurse guidance)

The focus on privacy stemmed from how it contrasted to a normal cervical cancer screening, which women feared for various reasons. For example, some feared that the gynaecologic examination would be painful,

It [ed. the self-sampling] is good. It’s not like the other test. [...] Everyone is afraid of the normal procedure because they say it’s painful. (Ester; assigned written/did not ask for nurse guidance).

Some also feared “the screening tools” and that the tools used for the gynaecologic examination would be unclean. Beatrice said that,

[...] by using the normal procedure, it is hard, [there are] so many people there. Everybody has diseases. Even though you are told the tools has been sterilized […] you are not sure.

(Beatrice; assigned written/asked for nurse guidance)

#### Nurse presence

Even though the women really liked the self-sampling, the majority of women did not believe in their own capabilities to conduct the sampling correctly, and therefore had a strong need for a health professional to be present in order to be comfortable doing it. Mary described it this way,

I am uneducated [...] I feel more comfortable [ed. when a nurse is present] because I believe that the nurses are professionals. [...] We were just doing it together. There was a cooperation between us – me and the nurse, and the nurses have a profession to do that. I felt comfortable doing it with her. (Mary; assigned written+/did not ask for nurse guidance).

The lack of trust in own capabilities also led to some women questioning the test result and that it may not be as reliable as a physician-collected sample,

I cannot believe that this test [ed. self-sampling] give the same results as the other one. I believe that [...] this self-sampling [...] is different from the nurse’s sampling, so the result will be different. (Mary; assigned written+/did not ask for nurse guidance).

However, after discussing their concerns with a nurse, they vanished. For example Patricia said that,

I was not sure about the results of this test [ed. self-sampling], but then I was explained [ed. by a nurse]. (Patricia; assigned written+/asked for nurse guidance).

#### Preferences

Despite some women experiencing symptoms while doing the self-sampling, 70% (*n* = 15) preferred self-sampling over a provider-based test for future screening examination, if given a choice. However, the women usually preferred to collect the self-sample with a nurse present,

I would prefer this one [ed. pointing at the self-sampling device] [...] because it’s easy and comfortable. (Lydia; assigned written+/asked for nurse guidance).

## Discussion

This qualitative study, which was conducted at the cervical cancer screening clinic at ORCI in Dar es Salaam, shows that the majority of participants liked self-sampling and preferred it over provider-based sampling despite experiencing difficulties while taking the self-sample. The women faced troubles in various steps during self-sample collection - no matter if they had been given only a written instruction or an oral and written instruction. Therefore, for the women to be comfortable in doing the self-sample, they needed a nurse to be present who could assist them when needed. Self-sampling was preferred over the normal screening procedure due to the women finding it more private compared to a standard cervical cancer screening, which involved a gynaecological examination that was associated with pain and fear of unclean screening tools. The results of the provider- and self-sample were concordant, and only two women were HPV-positive (9.5%), which is below the overall HPV-positivity rate in Tanzania of 20.9% [8]. However, the test results cannot be generalised outside the study setting giving the type of the study and sample size [39].

The findings on the feasibility of self-sampling are in line with a study from Rwanda, where women practiced doing the self-sampling with the presence of a clinician in order to maximise feasibility [24]. However, the results are somewhat in contrast to two randomised controlled trials from Nigeria and South Africa [25, 29]. In the semi-urban area of Nigeria, 185 out of 200 women returned the sample after conducting it at home, and the results were concordant with a physician-sample, which indicates that women were able to perform the sampling correctly on their own. In South Africa, where women were randomised to do either self-sampling at home or come to a clinic for provider-sampling and HPV testing, 47% of women conducted self-sampling at home and returned it, while 42% came to the clinic for sampling and testing. In addition, 30% of women in the “home group” were more likely to respond to the screening than those from clinic group [29]. This indicates that self-sampling may increase uptake of screening and that it may be feasible for women to conduct sampling at home despite not believing in their own capabilities as found in this study. It may also indicate that you may not need to follow the instruction 100% for the test be effective, though it is without the scope of this study to conclude anything on this speculation. Hence, more large-scale research is needed. However in line with our study, the RCT also found that whilst most women (60%) in the “home group” preferred to conduct the test at home due to it being more private and confidential, most women (71%) in the “clinic group” preferred doing it at the clinic mainly due to nurses being available to help [29].

Similar to other studies from Africa, this study found HPV self-sampling to be an acceptable screening method [20–29]. Further, other studies also report that women found self-sampling to be comfortable [20, 24, 26, 28, 29] and more private [25]. However, a study from Cameroon also found that the women did not trust the HPV test results from the self-sampling, and therefore preferred physician-sampling [32]. Despite African studies overall support our findings on acceptability, large-scale studies from other countries have found self-sampling to be feasible without a nurse being present [19–21]. Further research should be conducted to see if these findings hold in other areas of Tanzania.

### Limitations

This study has a number of limitations. Firstly, this is a pilot study using qualitative methods wherefore the sample is small. Hence, the results cannot be generalised outside the study’s setting. However, it provides detailed knowledge of what could challenge and improve the feasibility and acceptability of HPV self-sampling in an African context. Another sub-study of the CONCEPT project explores the feasibility and acceptability of HPV self-sampling quantitatively, and this study is partly influenced by some of the findings from this qualitative pilot study. The results are yet to be published, and though they will not provide as in-depth knowledge about the underlying mechanisms that influence feasibility and acceptability as this study, they will show if HPV self-sampling is feasible and acceptable on a larger scale. Further, due to the fact that the majority of the women had trouble with ‘breaking off the brush’, it could be relevant to evaluate the feasibility of other self-sampling devices that do not have this feature, in order to see if this could increase feasibility. However, it was without the scope of this pilot study to include another device. Yet, another self-sampling device has been chosen for the CONCEPT sub-study that evaluates HPV self-sampling quantitatively.

Another limitation of this study is that the perception of self-sampling was investigated among women who were already attending conventional cervical screening, hence, the findings may not reflect the general perception among Tanzanian women. Secondly, the study was conducted in an urban area, and the results may not be representative for other areas in Tanzania. Thirdly, the instruction guide could have influenced the women’s abilities to collect the self-sample. If the women had not understood the instruction guide properly this could be the reason why so many failed in doing the self-sample without nurse assistance. However, it was not our impression that the instruction guide was the main cause of why women did not feel certain of own capabilities in doing the test. Fourthly, we cannot rule out the occurrence of response bias though we judge this issue to be minimal as the women appeared to talk freely and openly during the interviews. Lastly, it may be relevant to pinpoint that a general limitation of HPV testing – both provider-based and self-sampling – is that it requires follow-up of the women who test HPV-positive, and findings from another CONCEPT sub-study shows that re-attendance at clinic level is challenging [40]. Hence, outreach screenings and HPV self-sampling at home level with follow-up at clinic level may be more promising than HPV testing at clinic level. However, according to the findings of this study, this would entail that a person who can explain the procedure would need to be present during the self-sampling, even if conducted at home. If this need is not taken into consideration, it could have a negative effect on the great potential of HPV self-sampling in a resource-limited setting like this. Yet it is highly likely that it would be difficult to conduct self-sampling at home with a nurse being present as it would be very labour intensive and because there is an overall shortage of health staff in Tanzania. Therefore, further research is needed of how best to implement HPV self-sampling and assess other alternatives of how to make women comfortable conducting self-samples outside a clinical setting, e.g. by training and qualifying other persons than nurses to support self-sampling outside a clinical setting.

## Conclusions

This study shows that HPV self-sampling is generally well-perceived among Tanzanian women who attend a patient-initiated screening. However, it is mainly feasible if supportive guidance is available. HPV self-sampling may be an important option within cervical cancer screening programs and it may have potential to increase screening coverage in Tanzania.

## Supplementary information

**Additional file 1.** Instructions for self-sampling of HPV specimen. Steps to be followed for HPV self-sampling (in English) used in the study

**Additional file 2 **The Interview Guide**.** Interview Guide used in the study

**Additional file 3 **Informed Consent form**.** Consent form signed by participants prior to self-sampling. Research checklist Research checklist**.** Complete SRQR checklist

## Data Availability

The data are available from the corresponding author on reasonable request.

## Abbreviations

ASRs: Age-standardised rates
CONCEPT: Comprehensive Cervical Cancer Prevention in Tanzania
HPV: Human Papillomavirus
HR: High-risk
KCMC: Kilimanjaro Christian Medical Centre
ORCI: Ocean Road Cancer Institute
VIA: Visual inspection with acid

## References

[1] Broutet N, O’Neal Eckert L, Ullrich A, Bloem P (2014). Comprehensive cervical Cancer control. A guide to essential practice.

[2] Ferlay J, Soerjomataram I, Dikshit R, Eser S, Mathers C, Rebelo M (2014). Cancer incidence and mortality worldwide: sources, methods and major patterns in GLOBOCAN 2012. Int J Cancer.

[3] Louie K, De Sanjose S, Mayaud P (2009). Epidemiology and prevention of human papillomavirus and cervical cancer in sub-Saharan Africa: a comprehensive review. Trop Med Int Health.

[4] Rahman R, Clark M, Collins Z, Traore F, Dioukhane E, Thiam H, Ndiaye Y, De Jesus E, Danfakha N, Peters K, Komarek T, Linn A, Linn P, Wallner K, Charles M, Hasnain M, Peterson C, Dykens J (2019). Cervical cancer screening decentralized policy adaptation: an African rural-context-specific systematic literature review. Glob Health Action.

[5] International Agency for Cancer Research. Tanzania, United Republic of. Source: Globocan, 2018 [Internet]: International Agency for Cancer Research; 2018. [cited 14 August 2019]. Available from: https://gco.iarc.fr/today/data/factsheets/populations/834-tanzania-united-republic-of-fact-sheets.pdf.

[6] Kahesa C, Kjaer S, Mwaiselage J, Ngoma T, Tersbol B, Dartell M (2012). Determinants of acceptance of cervical cancer screening in Dar es Salaam, Tanzania. BMC Public Health.

[7] Cunningham M, Skrastins E, Fitzpatrick R, Jindal P, Oneko O, Yeates K (2015). Cervical cancer screening and HPV vaccine acceptability among rural and urban women in Kilimanjaro region, Tanzania. BMJ Open.

[8] Dartell M, Rasch V, Kahesa C, Mwaiselage J, Ngoma T, Junge J (2012). Human papillomavirus prevalence and type distribution in 3603 HIV-positive and HIV-negative women in the general population of Tanzania. Sex Transm Dis.

[9] Runge AS, Bernstein M, Lucas AN (2019). Cervical cancer in Tanzania: a systematic review of current challenges in six domains. Gynecol Oncol Reports.

[10] Linde DS, Rasch V, Mwaiselage JD (2019). Competing needs: a qualitative study of cervical cancer screening attendance among HPV-positive women in Tanzania. BMJ Open.

[11] Katanga J, Kjaer SK, Manongi R (2019). Performance of careHPV, hybrid capture 2 and visual inspection with acetic acid for detection of high-grade cervical lesion in Tanzania: a cross-sectional study. PLoS One.

[12] Ngoma T, Muwonge R, Mwaiselage J, Kawegere J, Bukori P, Sankaranarayanan R (2010). Evaluation of cervical visual inspection screening in Dar Es Salaam, Tanzania. Int J Gynecol Obstet.

[13] Dartell M, Rasch V, Iftner T, Kahesa C, Mwaiselage J, Junge J, Gernow A, Ejlersen S, Munk C, Kjaer S (2014). Performance of visual inspection with acetic acid and human papillomavirus testing for detection of high-grade cervical lesions in HIV positive and HIV negative Tanzanian women. Int J Cancer.

[14] Untiet S, Vassilakos P, McCarey C, Tebeu P, Kengne-Fosso G, Menoud P (2014). HPV self-sampling as primary screening test in sub-Saharan Africa: implication for a triaging strategy. Int J Cancer.

[15] Ajenifuja O, Ikeri N, Adeteye O, Banjo A. Comparison between self sampling and provider collected samples for human papillomavirus (HPV) deoxyribonucleic acid (DNA) testing in a Nigerian facility. Pan Afr Med J. 2018;30. 10.11604/pamj.12/06/2018.30.110.14321. 10.11604/pamj.2018.30.110.14321 PMC6195243 30364362

[16] Bhatla N, Dar L, Patro A, Kumar P, Kriplani A, Gulati A, Iyer V, Mathur S, Sreenivas V, Shah K, Gravitt P (2009). Can human papillomavirus DNA testing of self-collected vaginal samples compare with physician-collected cervical samples and cytology for cervical cancer screening in developing countries?. Cancer Epidemiol.

[17] APROVIX (2019). *APROVIX*.

[18] Rovers Medical Devices (2019). *Devices Archive - Rovers Medical Devices*.

[19] Castell S, Krause G, Schmitt M, Pawlita M, Deleré Y, Obi N (2014). Feasibility and acceptance of cervicovaginal self-sampling within the German National Cohort (pretest 2). Bundesgesundheitsblatt Gesundheitsforschung Gesundheitsschutz.

[20] Jones H, Wiegerinck M, Nieboer T, Mol B, Westhoff C (2008). Women in the Netherlands prefer self-sampling with a novel Lavaging device to clinician collection of specimens for cervical Cancer screening. Sex Transm Dis.

[21] Igidbashian S, Boveri S, Spolti N, Radice D, Sandri M, Sideri M (2011). Self-collected human papillomavirus testing acceptability: comparison of two self-sampling modalities. J Womens Health.

[22] Ilangovan K, Kobetz E, Koru-Sengul T, Marcus E, Rodriguez B, Alonzo Y (2016). Acceptability and feasibility of human papilloma virus self-sampling for cervical Cancer screening. J Womens Health.

[23] Jones H, Brudney K, Sawo D, Lantigua R, Westhoff C (2012). The acceptability of a self-Lavaging device compared to pelvic examination for cervical Cancer screening among low-income women. J Womens Health.

[24] Ndayisaba G, Verwijs M, van Eeckhoudt S, Gasarabwe A, Hardy L, Borgdorff H (2013). Feasibility and acceptability of a novel Cervicovaginal lavage self-sampling device among women in Kigali, Rwanda. Sex Transm Dis.

[25] Bansil P, Wittet S, Lim J, Winkler J, Paul P, Jeronimo J (2014). Acceptability of self-collection sampling for HPV-DNA testing in low-resource settings: a mixed methods approach. BMC Public Health.

[26] Mitchell S, Ogilvie G, Steinberg M, Sekikubo M, Biryabarema C, Money D (2011). Assessing women's willingness to collect their own cervical samples for HPV testing as part of the ASPIRE cervical cancer screening project in Uganda. Int J Gynecol Obstet.

[27] Ogilvie G, Krajden M, van Niekerk D, Smith L, Cook D, Ceballos K (2016). HPV for cervical cancer screening (HPV FOCAL): complete round 1 results of a randomized trial comparing HPV-based primary screening to liquid-based cytology for cervical cancer. Int J Cancer.

[28] Rositch A, Gatuguta A, Choi R, Guthrie B, Mackelprang R, Bosire R (2012). Knowledge and acceptability of pap smears, self-sampling and HPV vaccination among adult women in Kenya. PLoS One.

[29] Jones H, Altini L, de Kock A, Young T, van de Wijgert J (2007). Home-based versus clinic-based self-sampling and testing for sexually transmitted infections in Gugulethu, South Africa: randomised controlled trial. Sex Transm Infect.

[30] Mahomed K, Evans D, Sauls C, Richter K, Smith J, Firnhaber C. Human papillomavirus (HPV) testing on self-collected specimens: perceptions among HIV positive women attending rural and urban clinics in South Africa. Pan Afr Med J. 2014;17. 10.11604/pamj.11/03/2014%20.17.189.3454. 10.11604/pamj.2014.17.189.3454 PMC4228994 25396015

[31] Mbatha J, Galappaththi-Arachchige H, Mtshali A, Taylor M, Ndhlovu P, Kjetland E (2018). Correction to: Self-sampling for human papillomavirus testing among rural young women of KwaZulu-Natal, South Africa. BMC Res Notes.

[32] Berner A, Hassel S, Tebeu P, Untiet S, Kengne-Fosso G, Navarria I (2013). Human papillomavirus self-sampling in Cameroon. J Low Genit Tract Dis.

[33] Modibbo F, Iregbu K, Okuma J, Leeman A, Kasius A, de Koning M (2017). Randomized trial evaluating self-sampling for HPV DNA based tests for cervical cancer screening in Nigeria. Infect Agents Cancer.

[34] Linde D, Andersen M, Mwaiselage J, Manongi R, Kjaer S, Rasch V (2017). Text messages to increase attendance to follow-up cervical cancer screening appointments among HPV-positive Tanzanian women (Connected2Care): study protocol for a randomised controlled trial. Trials.

[35] Mortensen G (2010). Drivers and barriers to acceptance of human-papillomavirus vaccination among young women: a qualitative and quantitative study. BMC Public Health.

[36] Scarinci I, Litton A, Garcés-Palacio I, Partridge E, Castle P (2013). Acceptability and usability of self-collected sampling for HPV testing among African-American women living in the Mississippi Delta. Women'sHealthIssues..

[37] Kvale S. Doing interviews. London: Sage Publications; 2007.

[38] O'Brien BC, Harris IB, Beckman TJ, Reed DA, Cook DA (2014). Standards for reporting qualitative research: a synthesis of recommendations. Acad Med.

[39] Viviano M, Tran P, Kenfack B, Catarino R, Akaaboune M, Temogne L, Tincho Foguem E, Vassilakos P, Petignat P (2018). Self- versus physician-collected samples for the follow-up of human papillomavirus-positive women in sub-Saharan Africa. Int J Womens Health.

[40] Linde D, Andersen M, Mwaiselage J, Manongi R, Kjaer S, Rasch V (2019). One-way text messages versus no text messages on attendance to follow-up cervical cancer screening among HPV-positive Tanzanian women (Connected2Care): a parallel-group randomized controlled trial (preprint).

