# Supplementary material for: “The best thing is that you are doing it for yourself” – perspectives on acceptability and feasibility of HPV self-sampling among cervical cancer screening clients in Tanzania: a qualitative pilot study
Source: BMC Womens Health. 2020 Mar 31;20:65. doi: 10.1186/s12905-020-00917-7 (PMC7110708; doi:10.1186/s12905-020-00917-7)
Supplement: Supplementary file 1 — Additional file 1. Instructions for self-sampling of HPV specimen. Steps to be followed for HPV self-sampling (in English) used in the study [file 12905_2020_917_MOESM1_ESM.pdf]

Additional file 1. Instructions for self-sampling of HPV specimen.

Steps to be followed:

- **Step 1: Preparation**

Client wash hands before the procedure. Remove the Qvintip self-collection device from the packaging by holding on the side of the blue grip. Extra cation should be taken to ensure the fingers do not touch the white portion of the device.

- **Step 2: Insertion**

Client in standing position should spread the legs and one leg stepped into an inclined position (Stairs/wooden bar). One hand of the client should spread the labia and the other hand inserts the Qvintip device into the vagina. Let the device slide into the vagina until it stops (~ 10cm).

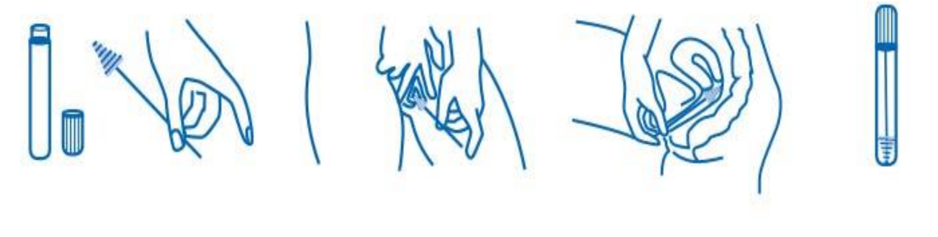

Figure I

- **Step 3: Collection of specimen**

Fully rotate the device twice (360°). Remove the device by holding it straight.

- **Step 4: Handling of device after specimen collection**

Place the Qvintip device on top the storing tube so that its white ends are directed towards the air (for at least 3 minutes). Preventing the white end from contacting anything or touched.

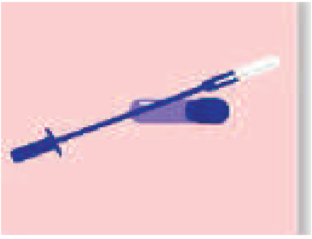

Figure II

- **Step 5. Placement of specimen into a container**

After the collections, place the specimen into the container. Bend the device against the container, pull a little bit, so that the white portion can fall into the container (Make sure the white portion is inside the container).

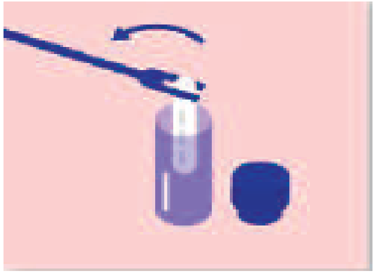

Figure III
