# Supplementary material for: “The best thing is that you are doing it for yourself” – perspectives on acceptability and feasibility of HPV self-sampling among cervical cancer screening clients in Tanzania: a qualitative pilot study
Source: BMC Womens Health. 2020 Mar 31;20:65. doi: 10.1186/s12905-020-00917-7 (PMC7110708; doi:10.1186/s12905-020-00917-7)
Supplement: Supplementary file 2 — Additional file 2 The Interview Guide. Interview Guide used in the study [file 12905_2020_917_MOESM2_ESM.pdf]

Study no: ………...

CONCEPT baseline no: ……..…………..

INTERVIEW GUIDE:

Feasibility and acceptability of HPV self-sampling in cervical cancer screening – a qualitative study in Tanzania.

Ocean Road Cancer Institute, Dar es Salaam, Tanzania
February – April 2017

FINAL VERSION - English

Date of interview: day [ ][ ] month [ ][ ] year [ ][ ][ ][ ]

Name of interviewer:	Participants initials:
Recording time:<br>Minutes [ ] Seconds [ ]	Name of translator:

Comments:

Introductory questions
Personal information's

1. How do you feel today? ……………………………………………………………………………………
2. How old are you? …………………………………
3. What is your marital status? ……………………………………………………………………………
   a. What does your husband do? ………………………………………………………………
4. Do you have kids? ………………………………………………………………………………………
   a. If yes, how many? What are their names? ……………………………………………….
5. What is your occupation? ………………………………………………………………………………
6. What is your religion? …………………………………………………………………………………
7. How many years have you gone to school? …………………………………………………………..
8. What is your favourite dish? …………………………………………………………………………….
9. What is you favourite colour? ………………………………………………………………………...

Transition

1. You have just been at the clinic today. Can you tell me what have you experienced there?
2. Why were you there? Why did you come to the clinic?
   a. Symptoms
   b. Did they help you in any way?

Opening question

1. The nurse gave you this test earlier on today [I am showing the test] and introduced you to a consent form. Can you tell me what exactly the nurse told you before you were asked to do the self-test?

2. What did you think when you looked at it?
   a. Did you wonder what is it for?

Key questions (Feasibility)

1. Can you describe to me, what did you do when the nurse gave you this test?

2. Can you describe to me the whole procedure of the self-collection? (What did you in the beginning? What did you do when you finished the test? Where did you put it? Did you give it to the nurse? Did you wash your hands?)

3. The nurse gave you also the instruction guide. Did you use it in any way? How did you use it?

4. How would you describe doing the test yourself? Easy/ Difficult

5. Was anything hard for you while doing this test?

6. What was the easiest during personal test?

Transition

Now, I would like to ask you a few questions about how your feelings about the personal test.

Key questions (Acceptability)

1. How did you feel during the personal test? Good/bad (Did you feel comfortable/uncomfortable, embarrassed, did you feel any pain or discomfort during the test, convenient vs. stressed)

2. Do you think that other women could be worried about something while doing this test?

3. If you could choose – would you prefer to do this test or having the nurse examining, you? Why?

4. It is possible to do this test in your own home instead of here at the clinic. How would you feel about doing this test at home? Would you prefer to do it with the nurse present? What do you think other women would prefer?

5. Do you think would it be easy to implement the self-test in your country? What would be an obstacle?

6. Do you think, that other women usually talk to their family/husband/friends about their health problems?

7. What was the best/the worst thing about the personal test?

8. Would you recommend personal test to other women? Friends/ Relatives.

9. If you could choose – is there anything you could change while doing this test?
(or in the instruction guide? The brush? The storage device? The handle?) Was the instruction guide understandable?

Closing questions

Thank you for being honest with me and for all your answers. Before we finish:

1. Is there anything else you would like to add?

………
……

I will turn off the recorder now (I do that before summary because the woman might open if the recorder is off –she will feel more comfortable).

But before we finish I would like to go through my notes and see if I understood everything correctly. I need a minute to do so, so please if there is anything you would like to add or to ask about, feel free.

Summary of the interview:

………
………
………
………
………
………
………
………
………
………
………
