# Supplementary material for: “The best thing is that you are doing it for yourself” – perspectives on acceptability and feasibility of HPV self-sampling among cervical cancer screening clients in Tanzania: a qualitative pilot study
Source: BMC Womens Health. 2020 Mar 31;20:65. doi: 10.1186/s12905-020-00917-7 (PMC7110708; doi:10.1186/s12905-020-00917-7)
Supplement: Supplementary file 3 — Additional file 3 Informed Consent form. Consent form signed by participants prior to self-sampling. Research checklist Research checklist. Complete SRQR checklist [file 12905_2020_917_MOESM3_ESM.pdf]

Ocean Road Cancer Institute (ORCI), Tanzania
University of Southern Denmark (SDU), Denmark

Study no: ....

CONCEPT baseline no: ……..………………….

CONTACT INFORMATION

(filled out by nurse)

NAME:

First:... Middle: .... Last: ....

RESIDENTIAL ADDRESS:

Region: .... Village: ....

District: .... Street name: ....

Ward: .... House no.: ....

Name of street leader/ popular person: ....

MOBILE PHONE NUMBER:

Primary: .... Secondary: ....

INFORMED CONSENT

Research description: You are invited to participate in the study that aims at better understanding of different methods in order to detect and improve cervical cancer prevention in your country. Cervical cancer is the most common cancer among women in Tanzania.

Procedure: Apart from regular screening – which is done by the nurse, you will participate in the personal test which you will do yourself. In order to help you to do the test – the written protocol will be given to you. Please follow the steps in the protocol. After doing the personal test, you will be interviewed by the researcher. She would like to hear and learn from you with regard to your experience with the test.

Benefit: The personal test can detect HPV infection – the main cause of cervical cancer. You will be contacted and treated if this infection will be confirmed.

Confidentiality: All your personal information's and answers will be anonymous, and not used in the study.

Voluntary participation: Your participation in the study is entirely voluntary.

Date **Participant´s signature**
